# Genomic analysis of antibiotic resistance genes and mobile genetic elements in eight strains of nontyphoid *Salmonella*

**DOI:** 10.1128/msystems.00586-24

**Published:** 2024-08-19

**Authors:** Haibing Liu, Lijie Zheng, Huimin Fan, Ji Pang

**Affiliations:** 1 Department of Clinical Laboratory, The Affiliated People’s Hospital of Jiangsu University, Zhenjiang, Jiangsu, China; University of California San Diego, La Jolla, California, USA

**Keywords:** nontyphoidal *Salmonella*, whole-genome sequencing, antimicrobial resistance gene, mobile genetic element

## Abstract

Nontyphoidal *Salmonella* (NTS) is the main etiological agent of human nontyphoidal salmonellosis. The aim of this study was to analyze the epidemiological characteristics and horizontal transfer mechanisms of antimicrobial resistance (AMR) genes from eight strains of NTS detected in Zhenjiang City, Jiangsu Province, China. Fecal samples from outpatients with food-borne diarrhea were collected in 2022. The NTS isolates were identified, and their susceptibility was tested with the Vitek 2 Compact system. The genomes of the NTS isolates were sequenced with the Illumina NovaSeq platform and Oxford Nanopore Technologies platform. The AMR genes and mobile genetic elements (MGEs) were predicted with the relevant open access resources. Eight strains of NTS were isolated from 153 specimens, and *Salmonella* Typhimurium ST19 was the most prevalent serotype. The AMR gene with the highest detection rate was AAC(6*'*)-Iaa (10.5%) followed by TEM-1 (7.9%), sul2 (6.6%), and tet(A) (5.3%). Eleven MGEs carrying 34 AMR genes were identified on the chromosomes of 3 of the 8 NTS, including 3 resistance islands, 6 composite transposons (Tns), and 2 integrons. Eighteen plasmids carrying 40 AMR genes were detected in the 8 NTS strains, including 6 mobilizable plasmids, 3 conjugative plasmids, and 9 nontransferable plasmids, 7 of which carried 10 composite Tns and 3 integrons. This study provided a theoretical basis, from a genetic perspective, for the prevention and control of NTS resistance in Zhenjiang City.

**IMPORTANCE:**

Human nontyphoidal salmonellosis is one of the common causes of bacterial food-borne illnesses, with significant social and economic impacts, especially those caused by invasive multidrug-resistant nontyphoidal *Salmonella*, which entails high morbidity and mortality. Antimicrobial resistance is mainly mediated by drug resistance genes, and mobile genetic elements play key roles in the capture, accumulation, and dissemination of antimicrobial resistance genes. Therefore, it is necessary to study the epidemiological characteristics and horizontal transfer mechanisms of antimicrobial resistance genes of nontyphoidal *Salmonella* to prevent the spread of multidrug-resistant nontyphoidal *Salmonella*.

## INTRODUCTION

Nontyphoidal *Salmonella* (NTS) is a gram-negative, facultatively anaerobic, nonspore-forming bacillus, belonging to the family Enterobacteriaceae, and is one of the commonest etiological agents of acute gastroenteritis disease globally. Most cases of gastroenteritis caused by NTS in humans are self-limiting diarrheal diseases and do not require treatment with antibiotics (1). However, invasive bacterial infections caused by NTS pose a particular threat to immunocompromised individuals, including those infected with human immunodeficiency virus 1 or malaria, infants, the elderly, and those with underlying comorbidities (2). If left untreated, infections with invasive NTS (iNTS) often result in meningitis, septicemia, and even death. NTS is reportedly responsible for 3.4 million enteric infections and over 680,000 associated deaths annually throughout the world (3). The areas with the highest incidence of iNTS disease are sub-Saharan Africa and Europe, with 227 and 102 cases of iNTS per 100,000 population, respectively (4). In China, the incidence of infectious NTS diseases was estimated to be 626.5 cases per 100,000 persons (5).

The emergence of bacteria with antimicrobial resistance (AMR) is a natural phenomenon that can be accelerated by the overuse of antibiotics and the action of selection pressures as well as the injudicious antimicrobial use by humans. The AMR of NTS is mainly caused by the misuse of antibiotics in animal agriculture, which is poorly regulated and defined (6). Many classes of antibiotics used to treat NTS infections in food-producing animals are the same as those used in humans, and drug-resistant NTS can be transmitted to humans via the farm-to-fork route. Between 2015 and 2018, the U.S. Centers for Disease Control and Prevention estimated that 16% of NTS were resistant to one or more antibiotics (7). The resistance rates of NTS to ampicillin (AMP), trimethoprim-sulfamethoxazole (SXT), ciprofloxacin (CIP), and ceftriaxone in China are 57.3%, 24.8%, 11.2%, and 9.9%, respectively (8). AMR is mainly mediated by resistance genes associated with enzymatic modification, energy-dependent efflux, target protection, and changes in the bacterial cell wall. Therefore, it is necessary to study the composition and distribution of the drug resistance genes of NTS to slow the development of AMR.

Mobile genetic elements (MGEs), including plasmids, integrons, phages, transposons (Tns), insertion sequences (ISs), integrative and conjugative elements (ICEs), and genomic islands, play key roles in the capture, accumulation, and dissemination of AMR genes among bacterial populations and in the evolution of bacterial genomes via conjugation, transduction, or transformation (9). The MGE-mediated horizontal transfer of foreign genes also contributes significantly to the evolution of NTS, including the acquisition of AMR and virulence genes. Wiesner et al. (10) described the IncA/C plasmids of a multidrug-resistant *S.* Typhimurium ST213 strain, which carried a plasmid-borne bla_CMY-2_ gene encoding an extended-spectrum β-lactamase, which could be transferred within bacterial communities by the conjugal machinery. The AcrAB-TolC efflux pump in *Salmonella* can be upregulated by the insertion of IS1 or IS10, leading to an increase in drug resistance (11).

Although NTSs that cause human infections are becoming increasingly resistant to antibiotics, there is currently no in-depth research into the composition and transmission of AMR genes in NTS in local areas of China. In recent years, whole-genome sequencing (WGS) techniques have been exploited to provide information about the complete genome of microorganisms, which can be analyzed by using bioinformatics tools to identify and provide deeper insights into molecular epidemiology typing, bacterial resistomes and virulence factors, and pan-genome plasticity mediated by MGEs (12). In this study, we sequenced the whole genomes of eight strains of NTS from outpatients in Zhenjiang City, Jiangsu Province, China, which caused acute gastroenteritis in humans, to analyze the compositions and distributions of their AMR genes and the MGEs associated with the horizontal transfer of these genes. The main objectives of this work were to provide a theoretical basis, from a genetic perspective, for the prevention and control of NTS resistance in Zhenjiang City.

## RESULTS

### Characterization of NTS isolates

In total, 153 specimens from patients with suspected acute food-borne gastroenteritis were collected, and 8 strains of NTS were isolated from 8 patients, including 6 children (<6 years old) and 2 elderly people (>65 years old), who were particularly at risk of acquiring iNTS disease. Serotype analyses indicated that Typhimurium was the most prevalent serotype among the eight strains (37.5%), and the serotypes of the remaining strains were Goldcoast, London, Enteritidis, Potsdam, and 1,4,[5],12:i:− (a monophasic variant of *S.* Typhimurium). The other related characteristics of the NTS isolates are shown in Table 1.

**TABLE 1:** The related characteristics of NTS isolates

Strain name	Sequence type	Serotype	Serovar antigen		
			Serum agglutination	SISTR^*a*^	SeqSero
XSK	ST19	Typhimurium	O4,12:Hi:H2	O1,4,[5],12:Hi:H1,2	O4:Hi:H1,2
CHC	ST19	Typhimurium	O4,12:Hi:H2	O1,4,[5],12:Hi:H1,2	O4:Hi:H1,2
ZCX	ST19	Typhimurium	O4,12:Hi:H2	O1,4,[5],12:Hi:H1,2	O4:Hi:H1,2
ZLQ	ST34	1,4,[5],12:i:−	O4,12:Hi	O1,4,[5],12:Hi	O4:Hi
YZY	ST2529	Goldcoast	O8:Hr:Hl,w	O6,8:Hr:Hl,w	O8:Hr:Hl,w
FFL	ST155	London	O3,10:Hl,v:H6	O3,10,15:Hl,v:H1,6	O3,10:Hl,v:H1,6
CYX	ST11	Enteritidis	O9,12:Hg,m	O1,9,12:Hg,m	O9:Hg,m
ZYX	ST2039	Potsdam	O7:Hl,v:He,n,z15	O6,7,14:Hl,v:He,n,z15	O7:Hl,v:He,n,z15

^*a*^ SISTR, *Salmonella In Silico* Typing Resource.

### Epidemiological surveillance of NTS isolates

**Fig 1:**
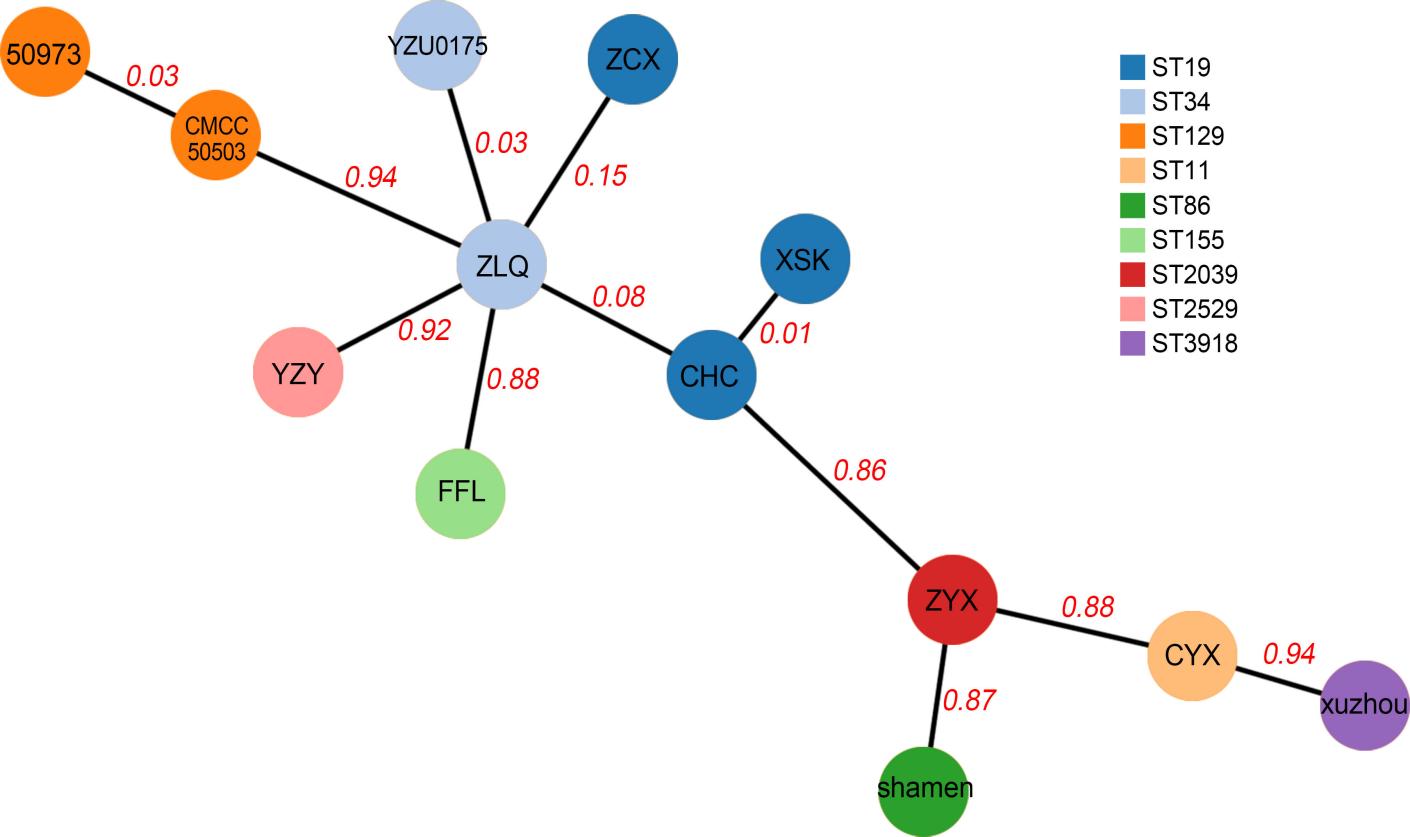
Minimum spanning tree of NTS isolates was constructed based on cgMLST.

To investigate the epidemiology of these 8 NTS strains, a minimum spanning tree of 13 strains of *Salmonella* was constructed based on core-genome multilocus sequence typing (cgMLST). As shown in Fig. 1, three strains of *S.* Typhimurium ST19 (XSK, CHC, and ZCX) shared the closest evolutionary relationship with *S.* Typhimurium ST34 YZU0175 (isolated from a diarrhea patient in Yangzhou City) and *S*. 1,4,[5],12:i:− ST34 ZLQ. No close evolutionary relationships were found for the remaining four NTS.

### Composition and distribution of AMR genes

**Fig 2:**
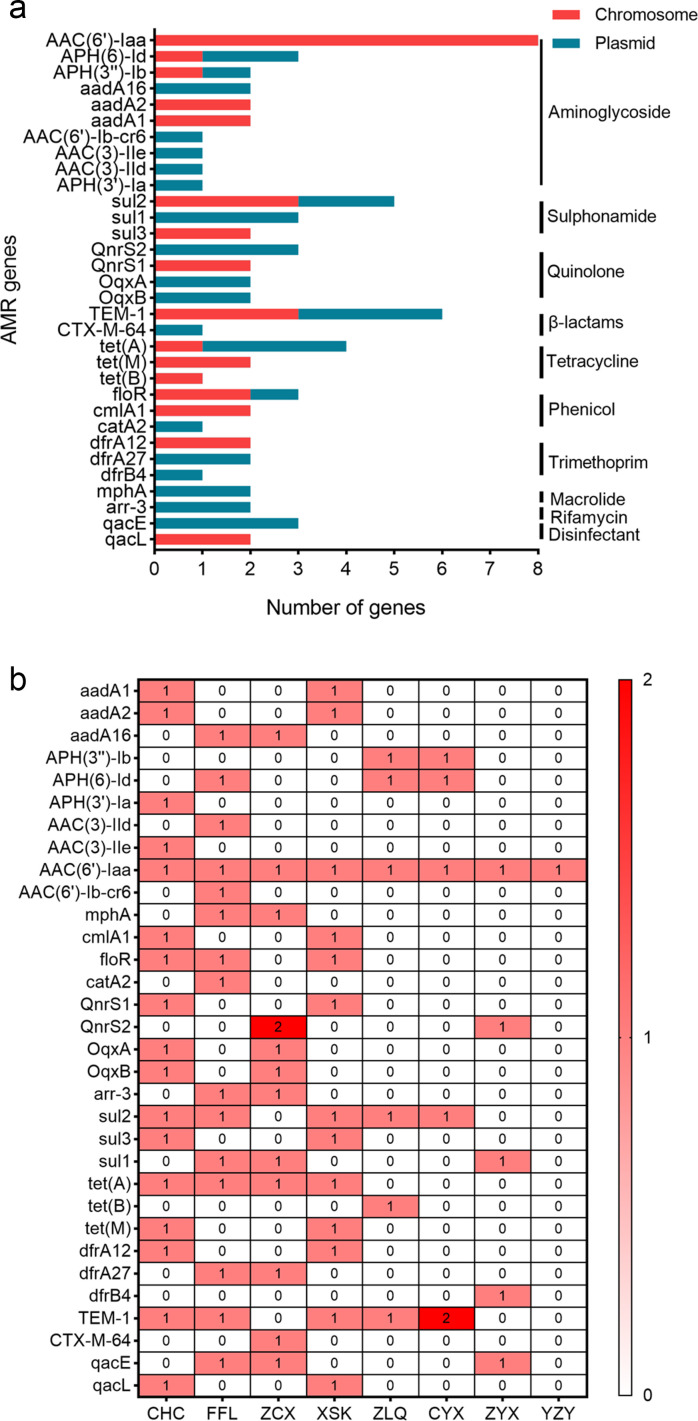
Composition and distribution of AMR genes. (a) Distribution of AMR genes located on chromosomes and plasmids of eight strains of NTS. (b) Heatmap of AMR gene distributions in eight strains of NTS.

A total of 76 acquired resistance genes were predicted, 40 of which (52.6%) were located on plasmids and the rest on chromosomes. The most prevalent AMR gene was AAC(6*'*)-Iaa (10.5%), encoding resistance to aminoglycosides, followed by TEM-1 (7.9%), encoding resistance to β-lactams; sul2 (6.6%), encoding resistance to sulfonamides; and tet(A) (5.3%), encoding resistance to tetracyclines. The antimicrobial category targeted by the most resistance genes was aminoglycosides (30.3%), followed by sulfonamides (13.2%), and β-lactams and tetracyclines (9.2%) (Fig. 2a). AMR genes were detected in eight strains of NTS, and *S.* Typhimurium CHC had the most AMR genes (17), followed by *S.* London FFL (15), *S.* Typhimurium ZCX (13), and *S.* Typhimurium XSK (13). Forty-three (56.6%) AMR genes were derived from 3 strains of *S.* Typhimurium, and 13 AMR genes were shared by *S.* Typhimurium XSK and CHC. The AMR gene profiles of *S*. 1,4,[5],12:i:− ZLQ and *S.* Enteritidis CYX were also similar (Fig. 2b).

The correlation between the resistance phenotypes (Table S1) and the AMR genes targeting the three classes of antibiotics recommended by Clinical and Laboratory Standards Institute (CLSI) was used to evaluate the ability of WGS to predict phenotypic resistance. Overall, phenotypic resistance correlated strongly with the presence of known AMR determinants. The overall sensitivity of the AMR genes detected in predicting resistance across the three classes of antibiotics was 100% (78.5%–100%), the specificity was 80% (49.0%–94.3%), and Kappa value was 0.8 (0.4–1.0). Among these genes, ampicillin-resistance-related genes had the strongest ability to predict the resistance phenotype (Table 2).

**TABLE 2:** Evaluation of the ability of WGS to predict resistance phenotypes^*a*^

Antibiotic	No. of test results				Sensitivity (%), (95% CI)	Specificity (%), (95% CI)	Kappa value, (95% CI)
	PT: R		PT: S				
	GT: R	GT: S	GT: R	GT: S			
AMP	6	0	0	2	100 (61.0–100)	100 (34.2–100)	1.0 (0.3–1.0)
CIP	4	0	1	3	100 (51.0–100)	75 (30.1–95.4)	0.75 (0.1–1.0)
SXT	4	0	1	3	100 (51.0–100)	75 (30.1–95.4)	0.75 (0.1–1.0)
Overall	14	0	2	8	100 (78.5–100)	80 (49.0–94.3)	0.80 (0.4–1.0)

^*a*^ R, resistant; S, susceptible; PT, phenotype; GT, genotype.

### Characterization of plasmids

A total of 18 plasmids were detected in 8 strains of NTS. Among these strains, *S.* Potsdam ZYX had the greatest number of plasmids (six), followed by *S.* Typhimurium CHC (four) and *S.* Enteritidis CYX, *S.* London FFL, and *S.* Typhimurium ZCX (two each). Of the 18 plasmids detected, 6 were identified as mobilizable plasmids, 3 as conjugative plasmids, and the remaining as nontransferable plasmids. A replicon typing analysis revealed that the mobilizable plasmids consisted of three Col(pHAD28) plasmids, two Col440I plasmids, and one ColRNAI plasmid. The conjugative plasmids included one IncHI2 plasmid, IncHI2A; one IncFIB(S) plasmid, IncFII(S); and one IncI1-I(Alpha) plasmid. A total of 40 AMR genes were identified across 8 plasmids, with 12 genes present in 2 conjugative plasmids and the remaining genes found in 6 nontransferable plasmids. The conjugative plasmids carrying AMR genes originated from *S.* Typhimurium ZCX, with pZCX-1 containing eight AMR genes and pZCX-2 containing four AMR genes. The pertinent details of these 18 plasmids are given in Table 3.

**TABLE 3:** The characterization of NTS plasmids

Plasmid name	Replicon type	Transferability	Antibiotic resistance genes	GenBank accession numbers
pCHC-1	p0111	No	tet(A)/ AAC (3)-IIe/ OqxA/ OqxB/APH(3')-Ia	OP927715
pCHC-2	ColRNAI	Mobilizable		OP927716
pCHC-3	Col440I	Mobilizable		OP927717
pCHC-4	Col(pHAD28)	Mobilizable		OP927718
pCYX-1	IncFIB(S) IncFII(S)	No	TEM-1	OP927719
pCYX-2	IncX1	No	TEM-1/sul2/APH(3'')-Ib /APH (6)-Id	OP927720
pFFL-1	IncI1-I(Alpha)	Conjugative		OP927721
pFFL-2	IncFIB(K)	No	sul1/qacE/aadA16/dfrA27/arr-3/AAC(6')-Ib-cr6/tet(A)/APH (6)-Id/TEM-1/AAC (3)-Iid/catA2/sul2/mphA/floR	OP927722
pXSK	Col440I	Mobilizable		OP927723
pZCX-1	IncHI2 IncHI2A	Conjugative	CTX-M-64/arr-3/dfrA27/aadA16/sul1/tet(A)/qacE/ mphA	OP927724
pZCX-2	IncFIB(S) IncFII(S)	Conjugative	QnrS2/QnrS2/OqxA/OqxB	OP927725
pZLQ	Col(pHAD28)	Mobilizable		OP927726
pZYX-1	IncU, pKPC-CAV1321	No	sul1/dfrB4/qacE	OP927727
pZYX-2	IncFIB(pECLA) IncFII(pECLA)	No		OP927728
pZYX-3	IncQ2	No	QnrS2	OP927729
pZYX-4	Col(pHAD28)	Mobilizable		OP927730
pZYX-5	Col440I	No		OP927731
pZYX-6	Col(pHAD28)	No		OP927732

### Analysis of MGEs carrying AMR genes located on chromosomes

**Fig 3:**
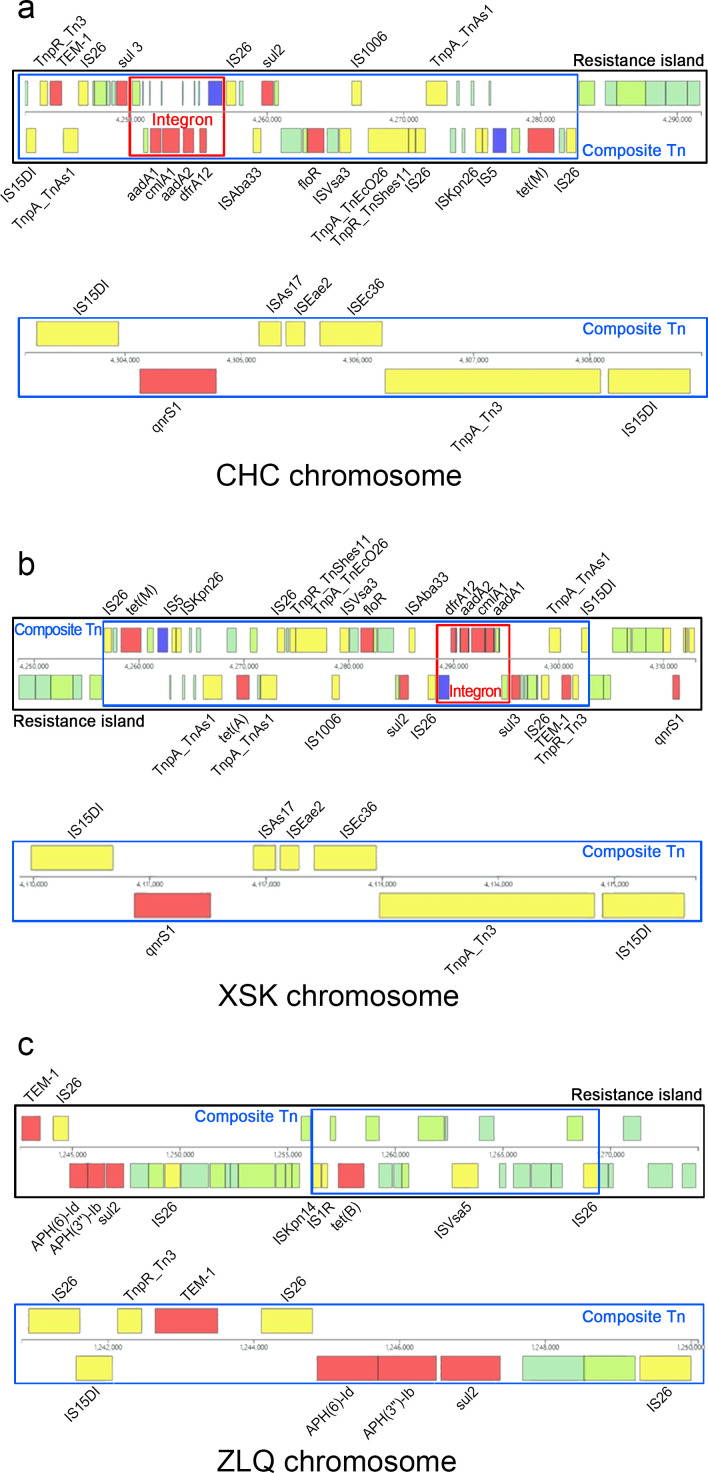
MGEs carrying AMR genes located on (a) chromosome of *S.* Typhimurium CHC; (b) chromosome of *S.* Typhimurium XSK; and (c) chromosome of *S*. 1,4,[5],12:i:− ZLQ.

A total of 11 MGEs carrying AMR genes were identified on the chromosomes of 3 of the 8 NTS: *S.* Typhimurium CHC, *S.* Typhimurium XSK, and *S*. 1,4,[5],12:i:− ZLQ. These MGEs included three resistance islands, six composite Tns, and two integrons. Specifically, the chromosome of *S.* Typhimurium CHC carried 1 resistance island, 2 composite Tns, and 1 integron, with a total of 10 AMR genes located within these MGEs. As shown in Fig. 3a, the primary structure of the resistance island was a composite Tn containing 15 ISs, 9 AMR genes, and 1 integron carrying 4 AMR genes. The chromosome of *S.* Typhimurium CHC contained another composite Tn flanked by IS15DI, which comprised six ISs and one QnrS1 gene. The MGEs associated with AMR genes carried on the chromosome of *S.* Typhimurium XSK showed similarities to those of *S.* Typhimurium CHC, except for an additional tet(A) AMR gene, one IS (TnpA_TnAs1), and three other genes presented in the composite Tn located in the resistance island (Fig. 3b). The chromosome of *S*. 1,4,[5],12:i:− ZLQ carried five AMR genes on two composite Tns: one containing four AMR genes and five ISs, and the other carrying a tet(B) gene and four ISs. These AMR genes and related MGEs constituted the primary structure of the resistance island (Fig. 3c).

### Analysis of MGEs carrying AMR genes located on plasmids

**Fig 4:**
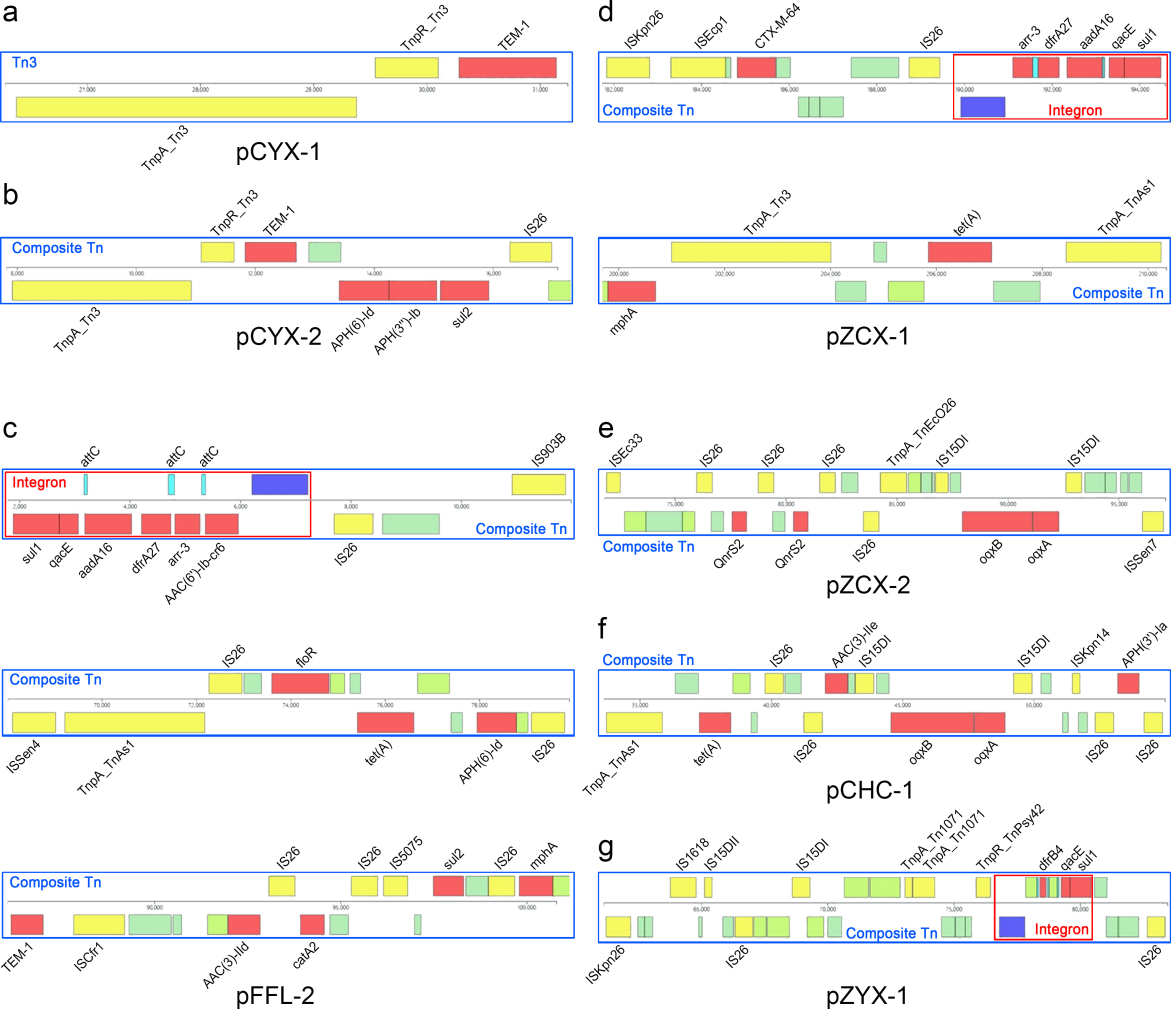
MGEs carrying AMR genes located on (a) pCYX-1, (b) pCYX-2, (c) pFFL-2, (d) pZCX-1, (e) pZCX-2, (f) pCHC-1, and (g) pZYX-1.

A total of 13 MGEs carrying AMR genes were identified on 7 plasmids and included 10 Tns and 3 integrons. The plasmid with the greatest number of MGEs was pFFL-2 (four), followed by pZCX-1 (three) and pZYX-1 (two). Nonmobile plasmids, pCYX-1 and pCYX-2, were isolated from *S.* Enteritidis CYX, and pCYX-1 carried a noncomposite Tn (Tn3) containing a TEM-1 gene (Fig. 4a), and pCYX-2 carried a composite Tn flanked by Tn3 and IS26, which harbored four AMR genes (Fig. 4b). The 14 AMR genes carried by pFFL-2 were distributed in 3 composite Tns, and a single integron containing 6 AMR genes was located in one of the composite Tns. A total of 10 ISs were involved in the formation of the 3 composite Tns, with IS26 accounting for 60% (Fig. 4c). The two conjugative plasmids isolated from *S.* Typhimurium ZCX, pZCX-1 and pZCX-2, contained three and one MGEs, respectively. The MGEs of pZCX-1 were two composite Tns and one integron, and eight AMR genes distributed across the two composite Tns (Fig. 4d). The MGE located in pZCX-2 was a composite Tn carrying four AMR genes, including two QnrS2 genes flanked by IS26, and the OqxA and OqxB gene flanked by IS15DI (Fig. 4e). The composite Tn located on pCHC-1 carried five AMR genes, which were distinct from those on the chromosome of *S.* Typhimurium CHC (Fig. 4f). The composite Tn present on pZYX-1 carried one integron, which harbored three AMR genes: dfrB4, qacE, and sul1 (Fig. 4g).

### Composition and distribution of ISs

**Fig 5:**
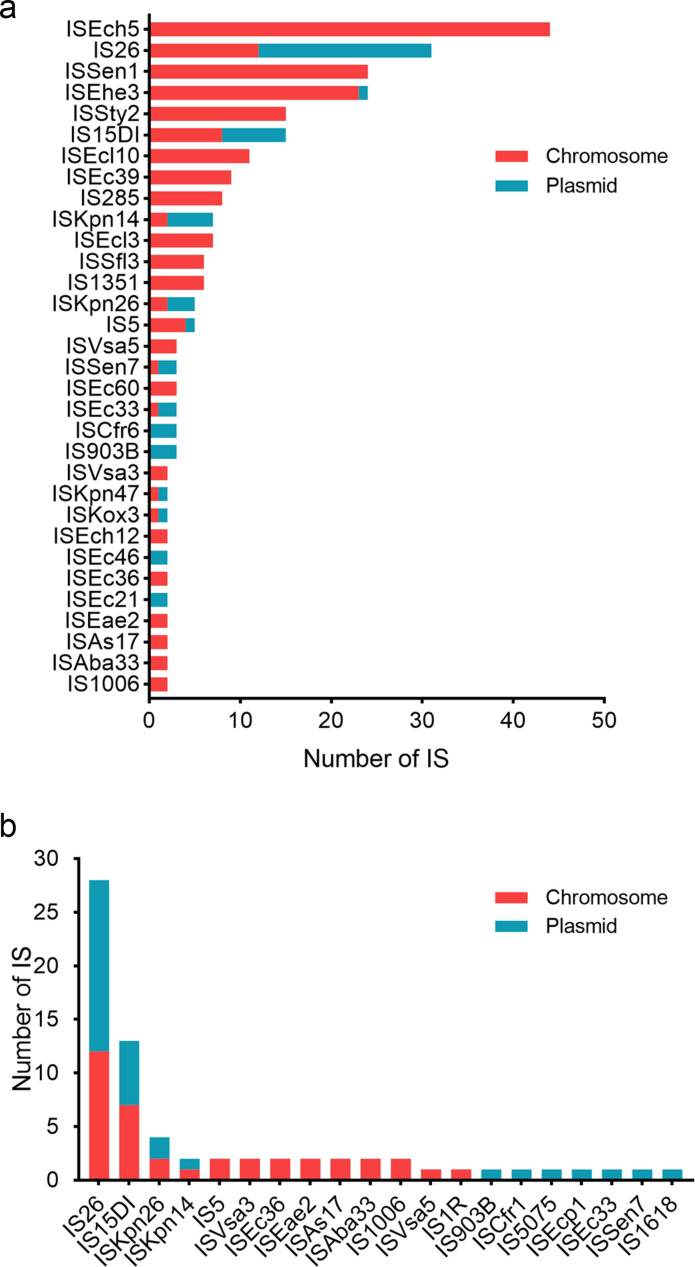
Composition and distribution of ISs identified in the eight NTS strains. (a) ISs located on chromosomes and plasmids. (b) Distribution of ISs involved in the formation of composite Tns.

A total of 277 ISs were identified in the 8 NTS strains, 211 of which were located on chromosomes and 66 on plasmids. The ISs with the highest detection rate were ISEch5 (15.9%), followed by IS26 (11.2%), ISEhe3 (8.7%), and ISSen1 (8.7%). ISEch5 and ISSen1 were only detected on chromosomes, and the plasmid-located IS with the highest detection rate was IS26 (Fig. 5a). Of the 277 ISs, 25% were involved in the formation of composite Tns, including 38 ISs located in composite Tns on chromosomes and 32 in composite Tns on plasmids. Among these, IS26 was the most numerous IS (28) that constituted composite Tns, followed by IS15DI (13) and ISKpn26 (4) (Fig. 5b).

### Identification of ICEs and integrative or mobilizable elements

**Fig 6:**
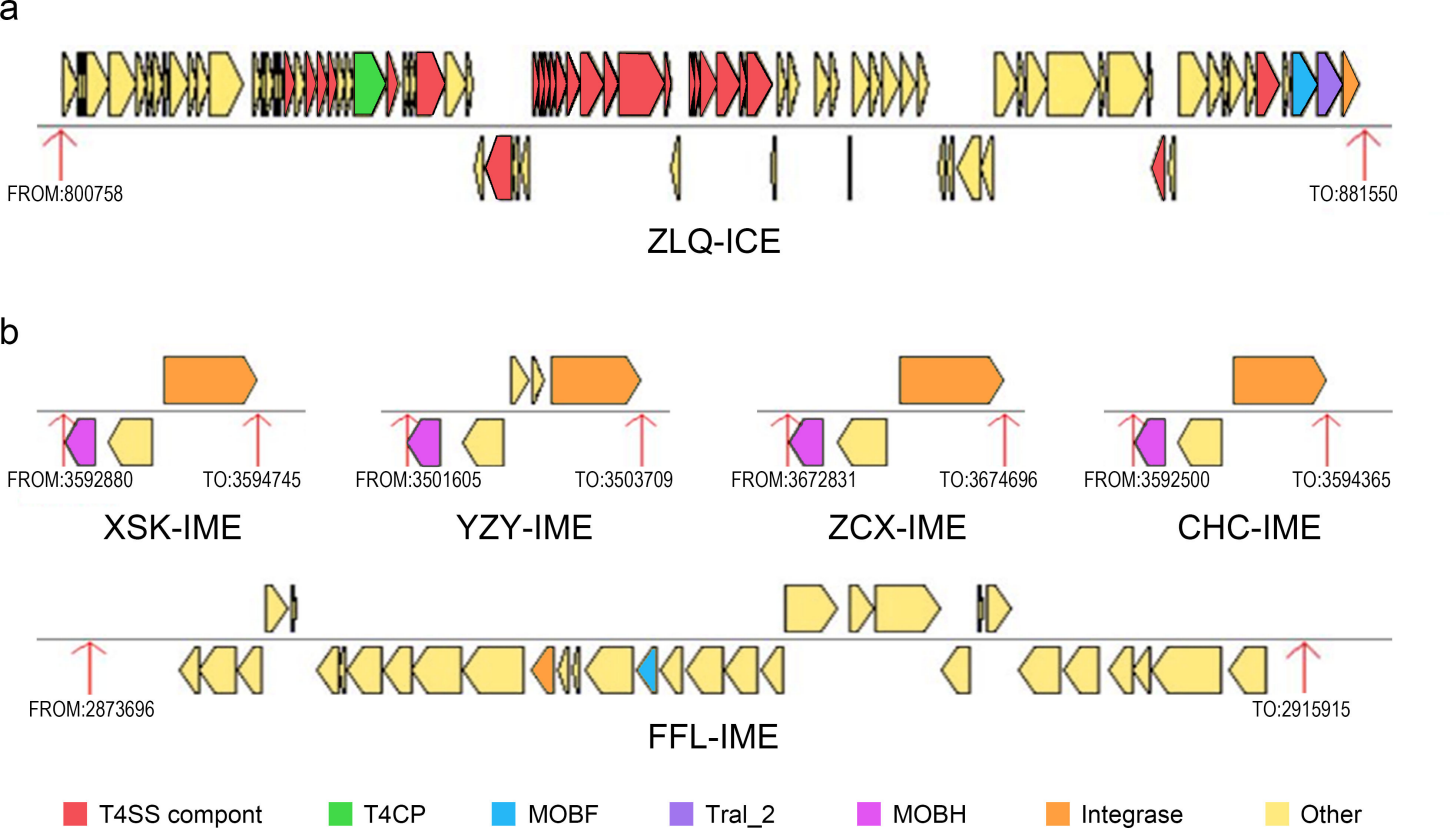
Identification of ICEs and IMEs. (a) ICE located on the chromosome of *S*. 1,4,[5],12:i:− ZLQ. (b) IMEs identified in five strains of NTS.

An ICE with a length of 80,793 bp was detected on the chromosome of *S*. 1,4,[5],12:i:− ZLQ, which was composed of 87 genes, including 24 genes related to the type IV secretion system, 2 genes encoding relaxase, 1 gene encoding integrase, 1 gene for the coupling protein to a type IV conjugative protein complex, and 59 genes encoding other proteins (Fig. 6a). A total of five integrative or mobilizable elements (IMEs) were identified in five NTS strains: *S.* Typhimurium CHC, *S.* Typhimurium XSK, *S.* Typhimurium ZCX, *S.* Goldcoast YZY, and *S.* London FFL. Each of these IMEs contained two important genes encoding integrase and relaxase, and the relaxase predominantly belonged to the MobH family (Fig. 6b).

### Analysis of prophages in NTS

**Fig 7:**
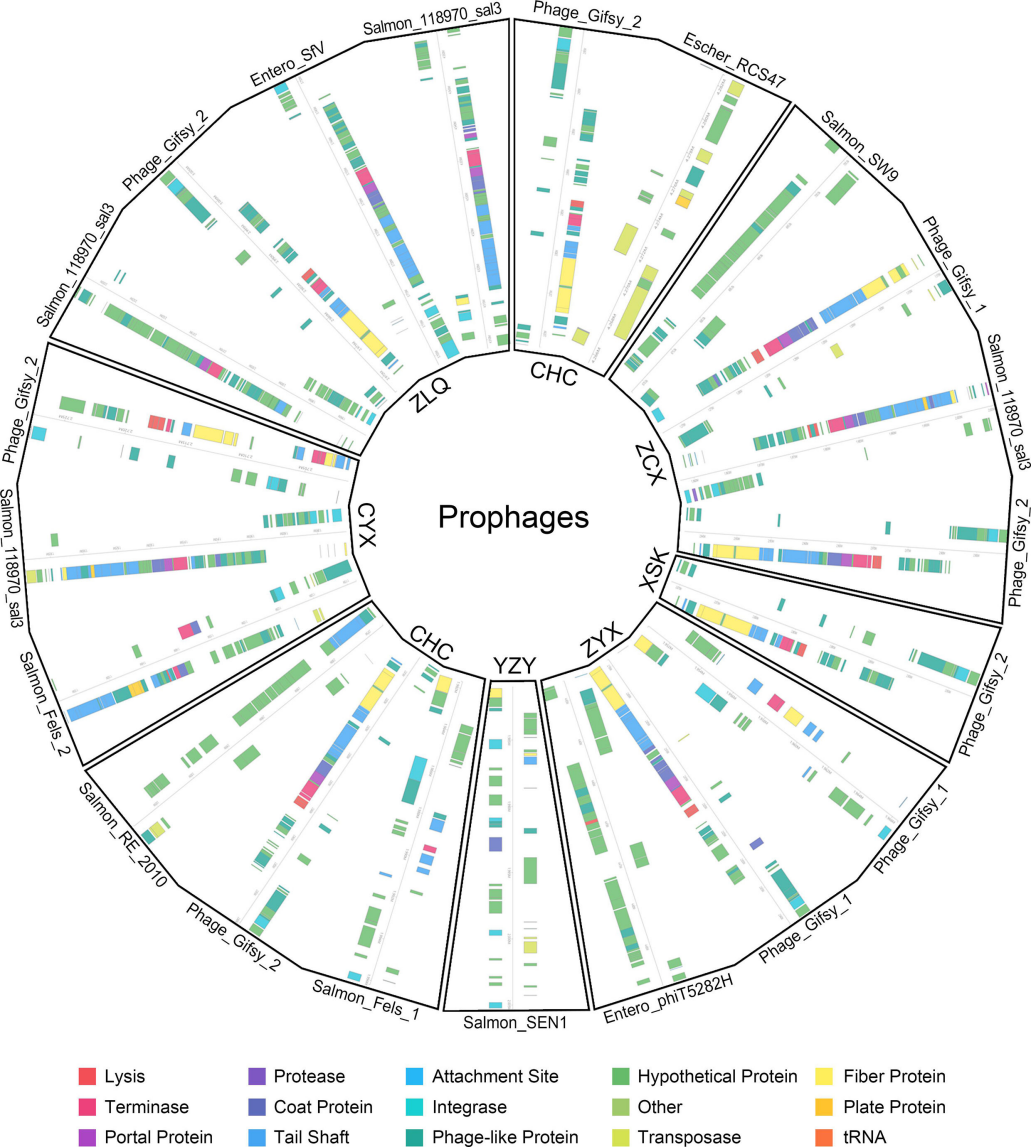
Prophage regions identified in eight strains of NTS.

A total of 21 intact prophage regions were detected in the 8 NTS strains, and the isolates with the greatest numbers of prophages detected were *S*. 1,4,[5],12:i:− ZLQ and *S.* Typhimurium ZCX (four each), followed by *S.* Potsdam ZYX, *S.* London FFL, and *S.* Enteritidis CYX (three each), *S.* Typhimurium CHC (two), and *S.* Goldcoast YZY and *S.* Typhimurium XSK (one each). Among these intact prophages, the most prevalent profiles were Phage_Gifsy_2 (six), Salmon_118970_sal3 (four), and Phage_Gifsy_1 (three), which were present in six, three, and two genomes, respectively (Fig. 7).

## DISCUSSION

Food-borne diseases are a serious public health problem in China, entailing considerable morbidity and mortality. In recent years, NTS has been recognized as the dominant pathogen of food-borne diseases in China, and *S.* Typhimurium and *S.* Enteritidis are the predominant serotypes, causing approximately 45.5% of NTS infections (13). Similar conclusions were drawn in our study, in that *S.* Typhimurium was the most prevalent serotype, accounting for 37.5% of the isolates identified. *S*. 1,4,[5],12:i:− is a monophasic variant of *S.* Typhimurium lacking the fljB-encoded second-phase H antigen, so *S*. 1,4,[5],12:i:− is closely related to *S.* Typhimurium. In this study, the close homology between *S*. 1,4,[5],12:i:− ZLQ and three strains of *S.* Typhimurium was also reflected in the epidemiology. We also found that a strain of NTS (*S.* Typhimurium YZU0175) with the same ST as *S*. 1,4,[5],12:i:− ZLQ caused diarrhea in a human from Yangzhou City, which is very close to Zhenjiang City.

Theoretically, any drug resistance phenotype of an organism can be derived from the AMR genotype. Therefore, it is necessary to study the composition and distribution of AMR genes to monitor and prevent multidrug-resistant NTS. In this study, a total of 76 AMR genes were predicted, including 71 determinants encoding resistance to 9 different antimicrobial categories and 5 determinants of disinfectant resistance. Most of the AMR genes targeted the antimicrobial categories of aminoglycosides, sulfonamides, β-lactams, and tetracyclines, and the most prevalent determinants in these categories were AAC(6*'*)-Iaa, sul2, TEM-1, and tet(A), respectively. Chen et al. reported that similar AMR gene patterns existed in NTS from Shaoxing City, in which 100% of isolates contained the AAC(6′)-Iaa_1 gene, 65.52% were positive for blaTEM−1B, and 52.87% contained the tet(A) gene (14). Predicting AMR genes with WGS technologies is an emerging technique for identifying drug-resistant bacteria to guide clinical treatment, although there are still many issues. For example, it is difficult to predict AMR accurately with WGS when it is caused by new genetic mutations, new resistance mechanisms, or the increased expression of intrinsic resistance genes (15). In this study, we found that identifying NTS strains clinically resistant to fluoroquinolones, AMP, or SXT with WGS was as reliable as conventional drug sensitivity testing, especially in predicting AMP resistance, for which 100% (61.0%–100%) sensitivity , 100% (34.2%–100%) specificity, and 1.0 (0.3–1.0) Kappa value were achieved. Our overall finding of strong correlations between phenotypic and genotypic resistance in *Salmonella* is consistent with the study of 640 strains of *Salmonella* from humans and retail meats, in which the resistance genotypes for AMP, CIP, and SXT correlated with the phenotypes with 99.7%, 100%, and 100% specificity, respectively, and 99.6%, 100%, and 86.4% sensitivity, respectively (16).

As important vectors in the transmission of AMR genes, MGEs play a very significant role in the evolution of NTS genomes, allowing them to adapt rapidly to selective pressures, especially in response to antimicrobial exposure. Transmissible plasmids, which have featured prominently as agents of the horizontal transfer of AMR genes in *Salmonella*, can be divided into two classes based on self-transmission: (i) conjugative plasmids, which contain the essential components for conjugation; and (ii) mobilizable plasmids, which must coexist in the donor bacterium with a conjugative plasmid to be mobilized by conjugation (17). In this study, the detection rate of transmissible plasmids was high, and they accounted for 50% of the total number of plasmids. The conjugative plasmids pZCX-1 and pZCX-2 are multiresistance plasmids carrying eight and three AMR genes, respectively, which make *S.* Typhimurium ZCX a high-risk strain for the transmission of AMR genes. The conjugative plasmid pFFL-1, which carries no AMR genes, coexisted with plasmid pFFL-2 in *S.* London FFL, and the 14 AMR genes carried by pFFL-2 could be transferred to pFFL-1 by composite Tns or integron to transmit by conjugation. No NTS with coexisting mobilizable plasmids and conjugative plasmids was detected in our study. Bacterial transposable elements are divided into four categories: ISs, composite Tns, Tn3 family, and prophages. In general, IS elements are the simplest autonomous mobile elements and can affect bacterial antibiotic resistance by gene inactivation or the transmission of AMR genes as part of a composite Tn. It has been reported that specific ISs composed of composite Tns correlate strongly with AMR genes. For example, ISKpn40 was present near all mcr-3 genes, and a strong association between IS30 and mcr-1.1 was observed (18). Similarly, there was an association between ISs and AMR genes in the NTS we identified. For example, the IS15DI-TnpR_Tn3-TEM-1-IS26 unit and IS15DI-QnrS1-ISAs17-ISEae2-ISEc36-TnpA_Tn3-IS15DI composite Tn were present in the chromosomes of *S.* Typhimurium XSK and *S.* Typhimurium CHC, and the IS15DI-TnpR_Tn3-TEM-1-IS26 unit was also detected on the chromosome of *S*. 1,4,[5],12:i:− ZLQ. Modules composed of specific ISs and AMR genes were identified in multiple plasmids, including the APH (6)-Id-APH(3*''*)-Ib-sul2-IS26 unit and IS15DI-OqxB-OqxA-IS15DI unit. Transposons, which commonly act as the primary reservoirs of AMR genes, provide functionality for the transfer of bacterial DNA segments to another part of the genome. Composite Tns flanked by ISs are the main form of bacterial transposons, and the distribution of ISs and AMR genes located on chromosomal composite Tns differs from that of plasmid-borne composite Tns. This unbalanced distribution of ISs and AMR genes located in composite Tns was also confirmed in our study. For example, AMR genes, including AAC(6*'*)-Iaa, aadA1, aadA2, sul3, tet(M), and QnrS1, and ISs, including IS5, ISVsa3, ISEc36, and ISEae2, were detected only on chromosomes, whereas AMR genes, such as QnrS2, aadA16, and dfrA27, and ISs, such as IS903B, ISCfr1, and IS5075, were only present on plasmids. Prophage sequences are a major source of genomic variability, providing a large number of AMR genes, virulence genes, and toxin genes for recipient bacteria. *Salmonella enterica* genomes contain large numbers of prophage profiles characterized by a high degree of variability (19). In the present study, 11 types of prophages were identified, and Salmon_118,970_sal3 and Phage_Gifsy_2 were highly prevalent. These results are consistent with those of previous studies, which reported that Salmon_118,970_sal3 was the most prevalent prophage profile in *Salmonella* (20). Furthermore, no AMR genes were detected in these prophage profiles. It is well known that integrons play a key role in the transmission of AMR genes and the evolution of *S. enterica*, especially class I integrons, which are widely distributed in *S. enterica* isolates (21). The five integrons identified in the present study were also class 1 integrons, which are transferred across bacterial genomes as components of Tns. Our results also showed that the AMR genes carried by two chromosomal integrons were quite different from those carried by three plasmid integrons, and the aadA1, aadA2, cmlA1, and dfrA12 genes were mainly located in chromosomal integrons, whereas sul1, qacE, aadA16, dfrA27, and arr-3 genes were mainly present in plasmid integrons. Resistance islands, defined as genomic islands that contain multiple resistance determinants, have a significant impact on the dissemination of AMR by horizontal gene transfer (22). *Salmonella* genomic island 1 (SGI1) is the first resistance island found in *S. enterica* Typhimurium DT104 isolates and contributes significantly to antibiotic resistance by carrying a class 1 integron that contains five AMR genes (23). In the present study, three resistance islands were identified, and almost all chromosomal AMR genes were located in them. They also carried multiple MGEs, including class 1 integrons, composite Tns, and ISs. The three resistance islands carried a greater number of AMR genes than SGI1, so they are potentially important vectors for the horizontal transfer of chromosomal AMR genes in *Salmonella*.

### Conclusions

To date, few studies have investigated the composition characteristics and horizontal transfer mechanisms of AMR genes from NTS in Zhenjiang City. In the present study, the AMR genes and MGEs of eight NTS strains from Zhenjiang City were identified with WGS combined with a bioinformatic analysis. *Salmonella* Typhimurium ST19 was the leading cause of nontyphoidal salmonellosis in Zhenjiang City. The most prevalent AMR genes included AAC(6*'*)-Iaa, TEM-1, sul2, and tet(A), which undergo horizontal gene transfer mainly on plasmids, composite Tns, and integrons. This study was limited by the low detection rate of NTS. Despite this, our study provides comprehensive information to better manage and prevent the spread of NTS AMR in Zhenjiang City.

## MATERIALS AND METHODS

### Sample collection and NTS identification

During 2022, fecal samples were collected from outpatients with food-borne diarrhea in Zhenjiang City, whose clinical presentations included diarrhea, vomiting, fever, nausea, and abdominal cramps. Patients taking any type of antibiotic were excluded from the study. Bacterial cultures were established by inoculating *Salmonella-Shigella* agar (CHROMagar) with the fecal specimens within 1 h of receiving them and incubating them at 37°C for 18–24 h. Presumptive colonies were further screened by testing in triple sugar iron agar, and the isolates were identified with the Vitek 2 Compact system (bioMérieux). The flagellar and lipopolysaccharide antigens were determined by slide agglutination with commercial antiserum (Ningbo Tianrun Bio-Pharmaceutical Co. Ltd), and the serotypes were assigned according to the Kauffmann-White scheme (24).

### Antimicrobial susceptibility testing

The antibiotics recommended by the Clinical and Laboratory Standards Institute for the treatment of enteric *Salmonella* infections include fluoroquinolones, AMP, and SXT. Therefore, the antimicrobial susceptibility of the NTS isolates for these three classes of antibiotics was tested with the Vitek 2 Compact system (bioMérieux), and the tested antimicrobial agents were AMP, CIP, and SXT. The results were interpreted based on the CLSI criteria (2022). Intermediate phenotypes were counted as resistant in this analysis. *Escherichia coli* ATCC 25922 was used as the control strain.

### Genomic analysis of AMR genes and MGEs

The NTS isolates were cultured in nutrient broth (Hangzhou Microbial Reagent Co. Ltd) for 24 h at 36°C, collected, and immediately transported to a commercial laboratory (Personalbio) for sequencing with the Illumina NovaSeq platform and the Oxford Nanopore Technologies (ONT) platform. Genomic analysis of AMR genes and MGEs mediated by WGS technology is divided into two parts as dry lab and wet lab. The term dry lab denotes the bioinformatic analysis, whereas the wet lab is related to molecular biology practical experiments (25).

#### Wet lab section

The genomic DNA was extracted with the CTAB method. The library for next-generation sequencing was constructed according to the standard preparative procedure of the Illumina TruSeq DNA LT Sample Preparation Kit, and the quality of the library was assessed with an Agilent Bioanalyzer 2100 according to the instructions of the Agilent High Sensitivity DNA Kit (Agilent Technologies Inc.). The QuantiFluor dsDNA System (Promega) was used for the quantitative analysis of the library according to the standard protocol of the Quant-iT PicoGreen dsDNA Assay Kit (Invitrogen). Paired-end sequencing was performed when the library met the quality requirements. The concentration of the genomic DNA used to construct a three-generation sequencing library was assessed with the Qubit dsDNA HS Assay Kit (Invitrogen), after which its purity was evaluated with a NanoDrop 2000 spectrophotometer (Thermo Fisher Scientific). Finally, the integrity of the DNA was confirmed with 0.35% agarose gel electrophoresis. The construction of the three-generation sequencing library, the assessment of its quality, and its sequencing were performed according to the standard protocols provided by ONT.

#### Dry lab section

The quality control of the raw reads for next-generation sequencing was performed with FastQC (26), adapter contamination was removed with Adapter Removal (version 2) (27), and all the reads were quality corrected with SOAPec (version 2.0) (28). The three-generation sequencing data were assembled with Unicycler (29) and Flye (30), and the assembled contigs were corrected with high-quality second-generation sequencing data using Pilon v1.18 (31). The serotypes of the NTS strains were predicted with the web-based tool SeqSero2 (v1.2.1) (32) and the *Salmonella In Silico* Typing Resource (v1.1.1) (33). The sequence type (ST) was identified with multilocus sequence typing (v2.22.0) (34). The publicly available resistance gene database ResFinder 4.4.2 was used to predict acquired resistance genes, with a threshold of 90% (35). The replicon type and the transferability of plasmids were determined with PlasmidFinder (v2.1.6) (36) and oriTfinder 1.1 (37), respectively. VRprofile 2 (38) was used to identify the MGEs carrying AMR genes, including integrons, composite Tns, and resistance islands. The MGEs that did not carry AMR genes were predicted with other open access resources, including IS elements with Isfinder (39), ICEs and IMEs with ICEberg (40) (with the BLAST default parameters), and prophages with PHASTER (41) (only “intact” prophages were used in this analysis). The minimum spanning tree was constructed with PHYLOViZ online (42) based on core-genome multilocus sequence typing, and five strains of *Salmonella* isolated from humans in Jiangsu Province were also used in the epidemiological analysis.

### Evaluation of the ability of WGS to predict phenotypic resistance

The ability of WGS to predict phenotypic resistance was evaluated by evaluating the correlation between the resistance phenotypes and AMR genes. When the phenotypic results were used as the reference standard, sensitivity was equal to the number of genotypically resistant strains divided by the total number of isolates showing phenotypic resistance, and specificity was equal to the number of isolates that were genotypically susceptible divided by the total number of strains with susceptible phenotypes. Correlation between phenotypic and genotypic antibiotic resistance was determined using Cohen’s Kappa coefficient of agreement and 95% confidence interval (CI) values. The kappa value was interpreted as follows: values of 0.81–1.00 indicated almost perfect agreement, values of 0.61CI0.80 indicated substantial agreement, values of 0.41CI0.60 indicated moderate agreement, values of 0.21CI0.40 indicated fair agreement, and values of ≤0.20 indicated slight to no agreement (43). Sensitivity, specificity, Kappa value, and 95% CI were calculated using OpenEpi version 3.01 (44).

## SUPPLEMENTAL MATERIAL

Table S1 msystems.00586-24-s0001.pdf Antibiotic resistance patterns of eight strains of nontyphoidal *Salmonella*.

Table S2 msystems.00586-24-s0002.xlsx Gene profiles for *Salmonella* chromosome.

ASM does not own the copyrights to Supplemental Material that may be linked to, or accessed through, an article. The authors have granted ASM a non-exclusive, world-wide license to publish the Supplemental Material files. Please contact the corresponding author directly for reuse.
