# Supplementary material for: Genomic analysis of antibiotic resistance genes and mobile genetic elements in eight strains of nontyphoid Salmonella
Source: mSystems. 2024 Aug 19;9(9):e00586-24. doi: 10.1128/msystems.00586-24 (PMC11406962; doi:10.1128/msystems.00586-24)
Supplement: Table S1 — Antibiotic resistance patterns of eight strains of nontyphoidal Salmonella. [file msystems.00586-24-s0001.pdf]

TABLE S1 Antibiotic resistance patterns of eight strains of nontyphoidal *Salmonella*

Strain name	Ampicillin		Ciprofloxacin		Trimethoprim-sulfamethoxazole	
	MIC	Explanation	MIC	Explanation	MIC	Explanation
XSK	≥32	R	0.5	I	≥320	R
CHC	≥32	R	2	R	≥320	R
ZCX	≥32	R	2	R	≥320	R
ZLQ	≥32	R	≤0.25	S	≤20	S
YZY	≤2	S	≤0.25	S	≤20	S
FFL	≥32	R	≤0.25	S	≥320	R
CYX	≥32	R	≤0.25	S	≤20	S
ZYX	≤2	S	2	R	≤20	S

MIC, Minimal inhibitory concentration; R, Resistant; I, Intermediate; S, Susceptible
